# Validation of an Ear-Worn Wearable Gait Analysis Device

**DOI:** 10.3390/s23031244

**Published:** 2023-01-21

**Authors:** Chang Keun Jung, Jinkyuk Kim, Hye Chang Rhim

**Affiliations:** 1 Beflex Research Center, Beflex Inc., Seoul 06628, Republic of Korea; 2 Department of Physical Medicine and Rehabilitation, Harvard Medical School, Spaulding Rehabilitation Hospital, Charlestown, MA 02129, USA; 3 Foot & Ankle Research and Innovation Lab (FARIL), Department of Orthopaedic Surgery, Harvard Medical School, Massachusetts General Hospital, Boston, MA 02115, USA

**Keywords:** gait analysis, running parameter, walking parameter, wearable sensor, impact force

## Abstract

Wearable devices capable of measuring gait parameters may provide a means to more economical gait analysis compared to conventional equipment comprising of a motion capture system and a forced treadmill. Beflex Coach (Beflex, Republic of Korea) is one such device but worn on the ear as Bluetooth earphones, unlike other wearables worn on the wrist, feet, or torso. In this study, the validity of the device was examined against a motion capture system and a forced treadmill for walking and running parameters. Five walking parameters (cadence, single support time, double support time, vertical oscillation (VO), and instantaneous vertical loading rate (IVLR)) and six running parameters (cadence, stance time, flight time, peak force, VO, and IVLR) were studied. Twenty young adults participated in walking or running on a forced treadmill at different speeds (walking: 0.8, 1.25, and 1.7 m/s for walking; running: 2, 2.5, and 3 m/s) while the two systems operated simultaneously. As a result, all parameters showed excellent associations (ICC > 0.75) and good agreements in Bland–Altman plots. The results of the study support the potential use of the ear-worn device as an inexpensive gait analysis equipment.

## 1. Introduction

Gait analysis is a systematic study for the characterization of human locomotion by measuring kinetic and temporal parameters [1]. Because gait involves complex coordination between the central nervous and musculoskeletal systems [2], its analysis can help monitor health status or estimate the severity of certain diseases. For example, analysis of walking gait can detect changes in spatio-temporal walking parameters such as increased stance duration, shortened step lengths [3], and reduced speeds [4], which have been related with aging and can even classify levels of frailty in the elderly [5]. Neurological disorders are also associated with gait abnormalities [6], and a previous study showed that the severity of Parkinson’s disease can be characterized by an increased percentage of double support duration relative to single support duration [7], a parameter that can be captured in gait analysis.

Furthermore, gait analysis has been employed in sports medicine and rehabilitation. Analysis of running gait mechanics may provide useful information for preventing running-related injuries [8] or even improving performance [9]. Previous studies reported that abnormal running mechanics may induce subsequent injuries [10,11] and suggested that maintaining appropriate running mechanics is important. Specifically, a high instantaneous vertical loading rate (IVLR) was associated with injuries involving stress fractures [12], and increased cadence [13] and decreased vertical excursion of the center of mass [14] were linked with improved performance and running economy. The IVLR, cadence, and center of mass are some parameters that can be characterized by gait analysis, and thus, such an analysis can aid in establishing running or running-involving sports with enhanced safety and efficiency.

However, setting up the environment to conduct gait analysis requires expensive equipment, such as motion capture cameras and force plates, and trained personnel [15]. Portable systems based on pressure sensors, such as F-scan^®^ (Tekscan, Boston, MA, USA), Pedar^®^ (Novel, St Paul, MN, USA), and inertial measurement unit sensors, such as e-AR (Sensixa, London, UK), Xsens MVN (Xsens, Enschede, The Netherlands), are also popular for gait analysis [16,17], but expensive and require highly skilled personnel to operate experiments and interpret measures. Therefore, gait analysis has been limited to clinicians [18], researchers [19], and professional athletes [20]. Moreover, gait analyses conducted in a lab-based setting are frequently considered artificial and may not capture natural gait [19].

Recently, wearable devices have shown the potential to provide inexpensive gait analysis. The advantages of wearables include increasing the accessibility of gait analysis and enabling it to be conducted in real-world settings [21]. Many wearable devices have been commercialized in different form factors and fixation locations [22]. The devices specialized for measuring gait parameters are mostly foot-mounted, waist-worn, and chest-worn [16] (see Section 4.2 for details). Similar to these devices, Beflex Coach (Beflex, Seoul, Republic of Korea) can measure gait parameters; however, it has the form factor of Bluetooth earphones, which is a relatively rare and new form. Thus far, no study has examined the validity of this device.

Therefore, this study aimed to evaluate the validity of the ear-worn wearable device, Beflex Coach, compared to a reference system (comprising a motion capture system and a forced treadmill) for estimating walking and running gait parameters.

## 2. Materials and Methods

### 2.1. Study Design

The present study aimed to evaluate the validity of Beflex Coach, an ear-worn wearable device, and compare the results with those of a reference system (comprising a motion capture system and a forced treadmill).

### 2.2. Participants

The eligibility of the participants for the study was based on self-reports on the satisfaction of the following inclusion criteria: (a) age between 19 and 35 years old, (b) no history of gait disorder due to musculoskeletal, neurological, or cognitive problems, (c) absence of running and walking problems. Informed consent forms were obtained from all individual participants included in the study.

### 2.3. Parameters

For parameter comparison, six running parameters and five walking parameters were investigated. The running parameters were four spatio-temporal parameters (cadence, stance time, flight time, and vertical oscillation (VO)) and two kinetic parameters (peak force and IVLR). The walking parameters were four spatio-temporal parameters (cadence, single support time, double support time, and VO) and one kinetic parameter (IVLR). The definitions and units of the gait parameters are listed in Table 1.

### 2.4. Procedures

**Figure 1:**
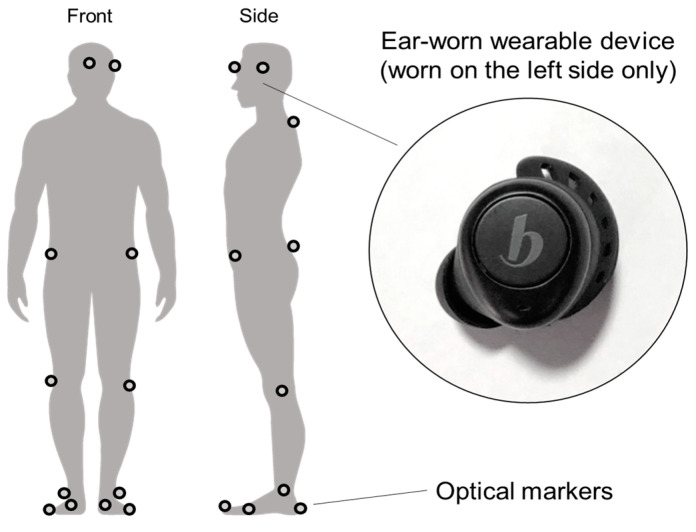
Ear-worn wearable device (Beflex Coach, Beflex Inc.) and optical marker placement locations.

In this study, Beflex Coach (Beflex, Seoul, Republic of Korea) was used as the target device for the validity investigation, and ten motion capture cameras (MotionAnalysis, Santa Rosa, CA, USA) and a split-belt treadmill with force plates (Bertec, Worthington, OH, USA) comprised the reference system. The wearable weighs 5.7 g, has a dimension of 17.1 × 20.2 × 22 mm, and has a price of 175,000 Korean won (around $137 US dollars). The ear-worn device was worn on the left ear of each participant (Figure 1), and the gait parameter data were collected from the device at 0.5 Hz. Subsequently, the gait data were transferred to the Beflex app (Beflex, Seoul, Republic of Korea)—the mobile app used to connect the device via Bluetooth—and uploaded to the server of the app. Finally, the exporting function of the app was used to collect the gait parameter data. The motion capture camera system tracked the three-dimensional (3D) location information of the 18 markers (second metatarsus, first and fourth metatarsals, heel, lateral ankle joint, lateral knee joint, left and right pelvis, sacrum, C8 joint, between eyebrows, and above left ear) that were attached to the participant (Figure 1). The force plates were set to collect the 3D ground reaction forces (GRFs) exerted at the left and right foot contacts. The sampling rates of the motion capture cameras and the forced treadmill were set at 400 Hz each. During the experiment, the participants were asked to walk or run at different speeds (walking speeds: 0.8, 1.25, 1.7 m/s, running speeds: 2, 2.5, 3 m/s), and each trial lasted for 2 min.

### 2.5. Data Process

**Figure 2:**
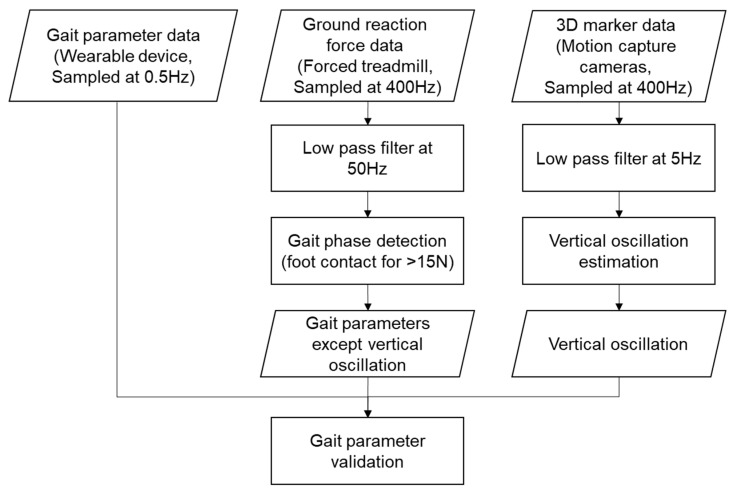
A flowchart showing the data processing steps for gait parameter validation.

The GRFs and the 3D marker location data were low-pass filtered at 50 Hz and 5 Hz, respectively, using separate sixth-order Butterworth filters. To exclude the transition states for acceleration and deceleration, the data acquired during the first and last 30 s were excluded from the analysis. For gait phase detection, a GRF of 15 N was used to determine the left and right foot contact and off phases. All reference gait parameter data, except the VO, were estimated from the GRF, and the VO was calculated as the mean vertical excursion distance of the left and right pelvis and the sacrum markers. The gait parameter data from the ear-worn device were simply obtained from the device output data. All parameters from each system were gathered from one minute of steady gait data, excluding the first and last 30 s, and were averaged for comparison. A flowchart of the data processing is shown in Figure 2.

### 2.6. Statistical Analysis

To examine the validity, intra-class correlation coefficients (ICCs) were calculated between the systems (Beflex versus the reference system) for all parameters. Similar to [23], a two-way random-effects model (ICC_2, K) was used for the validity determination. The agreement level between the systems was evaluated based on Bland–Altman plots, and the mean differences and limits of agreements were calculated. To examine the occurrence of heteroscedasticity of errors for each gait parameter, the coefficient of determination r^2^ was derived using the mean differences and the averages of the measurements from the two systems. Heteroscedasticity was considered to occur when r^2^ > 0.1 [24]. The statistical analysis in this study was conducted using MATLAB (MathWorks, Natick, MA, USA).

## 3. Results

### 3.1. Participants

Twenty young adults (ten males and ten females) participated and completed the study. The characteristics of the participants are listed in Table 2.

### 3.2. Validity

The ICC values, 95% confidence intervals, and *p* values for all gait parameters between the ear-worn wearable and the reference system are summarized in Table 3. Based on the interpretation of the ICCs in [25], all running and walking parameters obtained from the wearable device showed excellent association with the reference system (>0.75).

### 3.3. Agreement and Heteroscedasticity Test

**Figure 3:**
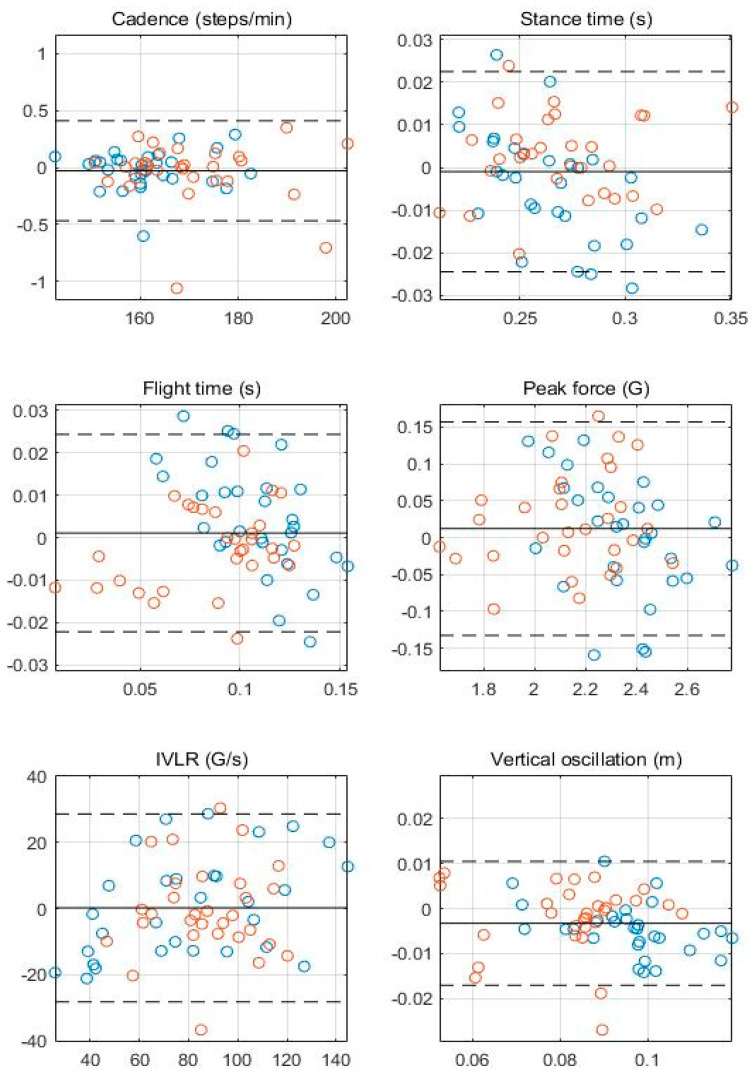
Bland−Altman plots comparing Beflex Coach and the reference system for running gait parameters. The bias is shown in a solid line, and the limits of agreements are shown in dashed lines. The average of the gait parameters from the two measurement systems are on the horizontal axes, and the relative differences between the systems are shown on the vertical axes. Each data point represents a trial from the twenty participants (blue and orange circles represent data points from males and females, respectively).

**Figure 4:**
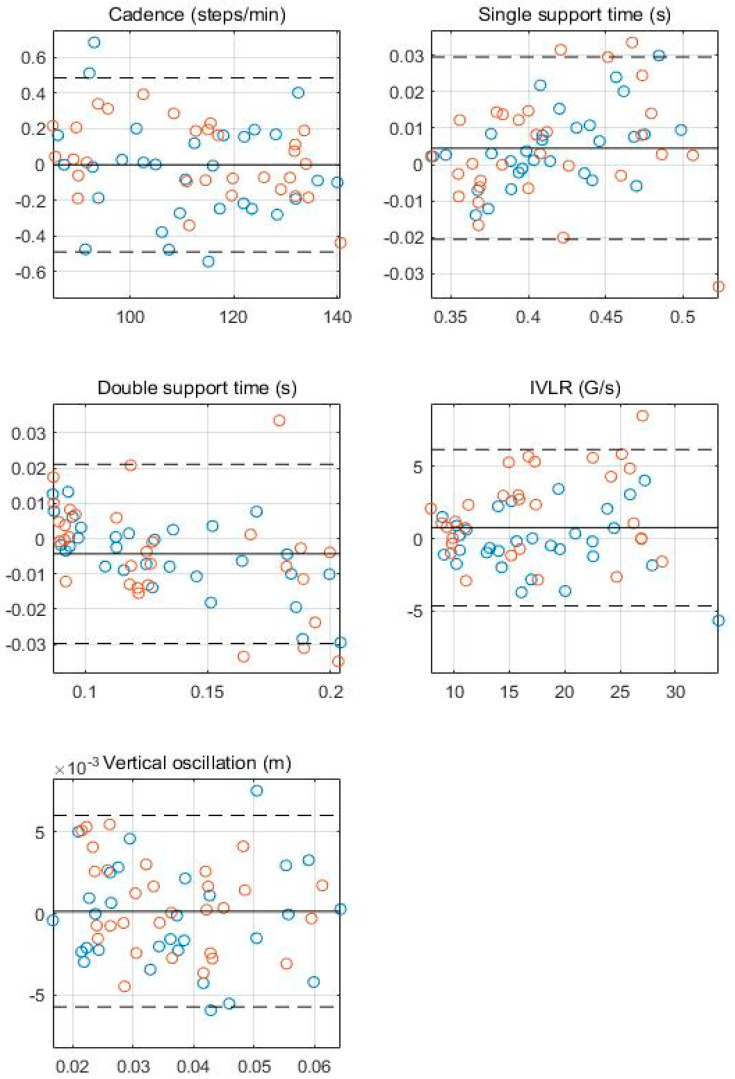
Bland−Altman plots comparing Beflex Coach and the reference system for walking gait parameters. The bias is shown in a solid line, and the limits of agreements are shown in dashed lines. The average of the gait parameters from the two measurement systems are on the horizontal axes, and the relative differences between the systems are shown on the vertical axes. Each data point represents a trial from the twenty participants (blue and orange circles represent data points from males and females, respectively).

In Figure 3, the Bland–Altman plots for all gait parameters between the ear-worn wearable and the reference system are shown. In Table 4, the mean difference, relative mean difference, limits of agreement, and coefficient of determination for each plot are listed. In Figure 3 and Figure 4, most of the values lie within the limits, supporting the agreement between the two systems. Weak heteroscedasticity of errors is observed for the double support time (r^2^ = 0.267), whereas it does not occur for all other parameters (r^2^ < 0.1), suggesting there are no systematic errors.

## 4. Discussion

In the current study, the validity of Beflex Coach was tested against a reference system comprising motion capture cameras and force plates. To evaluate the validity, the ICC values of six running (cadence, stance time, flight time, peak force, VO, and IVLR) and five walking (cadence, single support time, double support time, VO, and IVLR) parameters obtained using the two systems were derived. The results showed that all values ranged high (ICCs > 0.75), suggesting an excellent association between the two systems based on the interpretation of the ICCs in [25]. The Bland–Altman analysis yielded high degrees of agreement for all gait parameters, as reflected by their small mean differences and relative mean differences, and most of the trial data were within the upper and lower limits of agreement (LOA). To examine the occurrence of proportional errors between the systems, the coefficient of determination was calculated. No heteroscedasticity errors were found for all gait parameters except the double support time in walking. The results of our study suggest the potential of the ear-worn wearable as an economical gait analysis system for collecting the gait parameters considered in this study.

### 4.1. Potential Application of Ear-Worn Wearable Device

As the ear-worn device is equipped with speakers as in typical wireless earphones, if a real-time voice biofeedback program based on gait analysis data is integrated, the device may be utilized for gait retraining, a physical therapeutic method to facilitate healthy gait patterns [26]. In running studies, gait retraining was effective in reducing injuries and pain. The IVLR was correlated with lower limb stress fractures [27,28], and a significant reduction in its value was achieved when runners were gait-retrained [29,30]. Furthermore, a recent randomized controlled trial found that gait retraining was effective in lowering the IVLR, which, in turn, helped prevent running-related injuries. Specifically, there was a 62% reduction in the injury risk compared to the control group at the 12-month follow-up [30].

In neurological studies, intervention via biofeedback has been shown to alter walking gait patterns and alleviate symptoms. In a study in which subacute stroke patients were trained on treadmills, significant improvements were achieved in their spatiotemporal gait parameters (stance time, swing time, gait speed, and cadence), endurance, and mobility [31].

Although the efficacy of gait retraining based on biofeedback has been confirmed in numerous studies [32,33], 96% of studies were still limited to a laboratory environment [34]. The current device may expand gait retraining to in-field running exercises to prevent injuries and improve performance. Furthermore, the device could reduce the number of comprehensive lab-based analyses and provide an economical means for gait analysis in the daily environments of patients and older adults to promote and maintain healthy gait patterns.

### 4.2. Comparison with Other Wearable Devices

Many commercial wearables have been tested for the collection of gait parameters from various parts of the body: wrist, waist, chest, and feet.

Wrist-worn wearables such as Fēnix 2 (Garmin, Olathe, KS, USA) showed excellent correlation (0.931) for the cadence with motion capture cameras, and they are often used simultaneously with devices mounted on other parts of the body to collect extra parameters like vertical oscillation and stance time [35]. Foot pod devices such as Stryd (Stryd Inc., Boulder, CO, USA) and RunScribe (Scribe Lab. Inc., San Francisco, CA, USA) designed to be attached to shoelaces can provide various running parameters, including foot kinematics and kinetic and spatio-temporal parameters. The validity of the devices was also confirmed in [24], which reported excellent accuracies (all ICCs > 0.75) of the stance time, flight time, cadence, and step length compared to those derived using a high-speed camera. The pairing of the running dynamics of HRM-Run (GFR), a chest-mounted device (Garmin, Olathe, KS, USA), with a Garmin Forerunner 735XT (Garmin, Olathe, KS, USA) is also a promising method for gait analysis. The above chest-worn device was compared with Xsens MVN (Xsens Technologies B.V, Enschede, The Netherlands), a motion capture suit comprising a set of multiple inertial measurement units (IMUs). The concurrent validity (ICC) was found to be good for the VO (0.769), excellent for the cadence (0.970), and moderate for the stance time (0.568) [36]. There is also a waist-worn device, Myotest^®^ (Myotest, Sion, SA, Switzerland). In a study that compared the device to a high-speed camera, the validity of the former was determined as fair to excellent. The study obtained reasonable ICC values for the stance time (0.638 ± 0.106) and the flight time (0.692 ± 0.047) and an excellent ICC for the step frequency (0.885 ± 0.05) [37]. Compared to the other types of commercially available wearables, the ear-worn device examined in the current study showed comparable or better accuracy for all parameters and activity types, supporting its feasibility.

The ear has received relatively little attention as a potential location for wearable-based gait analysis. To our best knowledge, only one other ear-worn device has been developed: e-AR. However, this device is considered as an IMU sensor module instead of a consumer wearable device because it only provides raw motion data and not processed gait parameter data [38]. Thus, the studies utilizing this device first developed their own algorithms to extract gait parameters and subsequently compared the results with those obtained from other gait measurement systems. Atallah et al. derived kinetic and temporal parameters using e-AR and compared the parameters with those derived using a forced treadmill [39]. The study showed weak correlations between the kinetic parameters of the device and the reference system and the occurrence of proportional systematic errors in the temporal parameters. In a recent study, Diao et al. derived temporal parameters using the above ear-worn device and achieved good parameter estimations [40], supporting the potential of the ear area as a good location for a wearable attachment. Owing to the differences in the device outputs, the current study did not develop a gait parameter estimation algorithm; instead, it compared running parameters in addition to walking parameters. Compared to the previous studies, the current study yielded higher accuracy results as reflected by the smaller mean differences and limits of agreements of the temporal parameters.

### 4.3. Limitations

The results of the present study should be interpreted with caution owing to the following limitations. The study was conducted on young Korean participants, and the highest running speed was limited to 3 m/s. Therefore, the results of this study may not be generalized to other ethnic or age groups or running speeds beyond 3 m/s. Moreover, the trials were not repeated, and the reliability of the wearable device was not tested in the study.

## 5. Conclusions

The current study examined and confirmed the high validity and degree of agreement between an ear-worn wearable device (Beflex Coach) and a reference system (comprising a motion capture system and a forced treadmill) for estimating walking and running gait parameters. These results indicate that the ear-worn wearable device may provide a means to low-cost and portable gait analysis under conditions similar to those in the current study. If applied to gait retraining, the ear-worn wearable may expand previous gait treatment methods, which have been limited to indoor environments, to actual fields, benefitting diverse populations ranging from runners to older adults.

## Figures and Tables

**Table 1:** Definitions of gait parameters employed in the study. All parameters are averaged values of parameters obtained in each trial.

	Parameter	Definition	Unit
Running	Cadence	Number of steps taken per minute	steps/min
	Stance time	Time duration that one foot is in contact with the ground	s
	Flight time	Time duration that neither foot is in contact with the ground	s
	Peak force	Peak value of vertical ground reaction force	G
	IVLR	The steepest slope of vertical ground reaction force	G/s
	Vertical oscillation	Vertical displacement of center of mass between steps	m
Walking	Cadence	Number of steps taken per minute	steps/min
	Single support time	Time duration that either one foot is in contact with the ground	s
	Double support time	Time duration that both feet are in contact with the ground	s
	IVLR	The steepest slope of vertical ground reaction force	G/s
	Vertical oscillation	Vertical displacement of center of mass between steps	m

**Table 2:** Participant description. The mean values with standard deviations are shown. The median values are shown in brackets.

	n	Age, Years	Height, cm	Weight, kg
Male	10	25.2 ± 2.6 (25.5)	172.4 ± 8.0 (173.2)	68.4 ± 12.3 (75.3)
Female	10	27.2 ± 3.0 (27.0)	162.4 ± 6.4 (160.0)	55.6 ± 6.7 (53.1)

**Table 3:** Intra-class correlation coefficients and 95% confidence intervals (CI) between Beflex Coach and the reference system.

	Parameter	ICC	Lower CI	Upper CI	*p*
Running	Cadence (steps/min)	1.000	1.000	1.000	<0.001
	Stance time (s)	0.958	0.930	0.975	<0.001
	Flight time (s)	0.960	0.933	0.976	<0.001
	Peak force (G)	0.975	0.959	0.985	<0.001
	IVLR (G/s)	0.928	0.879	0.957	<0.001
	Vertical oscillation (m)	0.937	0.870	0.966	<0.001
Walking	Cadence (steps/min)	1.000	1.000	1.000	<0.001
	Single support time (s)	0.979	0.962	0.988	<0.001
	Double support time (s)	0.969	0.945	0.982	<0.001
	IVLR (G/s)	0.953	0.920	0.972	<0.001
	Vertical oscillation (m)	0.986	0.976	0.991	<0.001

**Table 4:** Mean difference, relative mean difference, limits of agreements, and coefficient of determination r^2^ between Beflex Coach and the reference system.

	Parameter	Mean Difference	Relative Mean Difference (%)	Lower LOA	Upper LOA	r^2^
Running	Cadence (steps/min)	−0.029	−0.02	−0.468	0.411	0.004
	Stance time (s)	−0.001	−0.37	−0.024	0.022	0.050
	Flight time (s)	0.001	1.11	−0.022	0.024	0.000
	Peak force (G)	0.012	0.54	−0.133	0.157	0.022
	IVLR (G/s)	0.183	0.22	−28.146	28.512	0.076
	Vertical oscillation (m)	−0.003	−3.68	−0.017	0.010	0.062
Walking	Cadence (steps/min)	−0.002	0.00	−0.491	0.486	0.055
	Single support time (s)	0.004	1.08	−0.021	0.030	0.068
	Double support time (s)	−0.004	−3.27	−0.030	0.021	0.267
	IVLR (G/s)	0.752	4.31	−4.635	6.139	0.009
	Vertical oscillation (m)	0.000	0.37	−0.006	0.006	0.012

## Data Availability

The data presented in this study are available on request from the corresponding author. The data are not publicly available due to privacy restrictions.

## Abbreviations

The abbreviations used in this manuscript are as follows (in the order of appearance):

IVLR	Instantaneous vertical loading rate
VO	Vertical oscillation
3D	Three-dimensional
GRF	Ground reaction force
ICC	Intra-class correlation coefficient
CI	Confidence interval
LOA	Limits of agreement
